# Psychological experience of Juvenile patients’ parents in Fangcang shelter hospital during the Omicron wave of COVID-19 pandemic in Shanghai, China: a qualitative study

**DOI:** 10.1186/s12889-022-14689-2

**Published:** 2022-11-28

**Authors:** Hongmei Wu, Kexi Liao, Caidie Yang, Nian Zhou, Heng Dou, Zhi Xu, Lingling Chu, Caiping Song, Chunmei Luo

**Affiliations:** 1 The First Unit, The Third Department, The First Hospital, National Exhibition and Convention Center (Shanghai) Fangcang Shelter Hospital, Shanghai, People’s Republic of China; 2 Respiratory and Critical Care Medical Center, Xinqiao Hospital, Army Medical University, 83 Xinqiao Main Street, Shapingba District, Chongqing, China; 3 Institute of Hepatobiliary Surgery, Southwest Hospital, Army Medical University, Chongqing, China; 4 Department of Nursing Administration, Xinqiao Hospital, Army Medical University, 83 Xinqiao Main Street, Shapingba District, Chongqing, China; 5 Office of Hospital Administration, Xinqiao Hospital, Army Medical University, 83 Xinqiao Main Street, Shapingba District, Chongqing, 400037 China; 6 Department of Orthopedics, Xinqiao Hospital, Army Medical University, 83 Xinqiao Main Street, Shapingba District, Chongqing, 400037 China

**Keywords:** Omicron, Fangcang shelter hospital, Juvenile patients, Juvenile patient’s parents, Psychological experience, COVID-19, Qualitative study

## Abstract

**Objective:**

To explore the psychological experience of Juvenile patient’s parents in Fangcang shelter hospital during the Omicron wave of COVID-19 pandemic.

**Methods:**

A qualitative study was conducted by using a phenomenological research method. Sixteen parents of juvenile patients with COVID-19 were recruited from National Exhibition and Convention Center (Shanghai, China) Fangcang shelter hospital (FSH) using purposive sampling. Data were collected by face-to-face in-depth interviews over 27 days, from April 9 to May 6, 2022. The interview data were analyzed using Colaizzi seven-step analysis method.

**Results:**

The psychological experiences of the parents of juvenile patients in the Fangcang shelter hospital were summarized into three themes: "perception regarding the FSH", "worried about the unmet needs of juvenile patients ", and "the psychological burden after discharge". These themes were classified into 9 sub-themes, including the acceptance of FSH, adaptability to FSH, concerns about cross-infection in the FSH, special needs of infants and young children, psychological needs of preschool children, the learning demands of school-age children, concern about re-positive, fear of sequelae, worry about social acceptance.

**Conclusion:**

Juvenile patients and their parents in the Fangcang shelter hospitals have both positive and negative experiences. It is suggested that facilities for minors should be planned in advance. Humanistic care for adolescent patients and health education for the public are also critical.

## Introduction

The Omicron variant SARS-COV-2 variant has quickly replaced the Delta variant as the dominant epidemic strain since the end of 2021. As of 10 October 2022, a total of 621,534,037 COVID-19 cases have been diagnosed with 6,557,720 deaths worldwide [1].

Omicron variant strains are characterized by high viral load, strong transmissibility, and concealability with a large number of asymptomatic carriers, posing a new threat to China's COVID-19 epidemic prevention and control [2, 3]. In late February, 2022, there was an outbreak of the Omicron wave of the COVID-19 pandemic in Shanghai.

To fight against this Omicron wave of COVID-19 pandemic, numerous Fangcang shelter hospitals (FSH) were established in Shanghai under the guidance of China's general policy of "dynamic zero COVID-19 strategy". FSH is a primary public health measure adopted by China during the critical period of epidemic prevention and control of major infectious diseases [4, 5]. The National Exhibition and Convention Center (Shanghai, China) FSH is the largest one in Shanghai with approximately 50,000 beds. From April 9 to May 6, 2022, a total of 12,357 infected patients were admitted to the No.3 hall of National Exhibition and Convention Center (Shanghai, China) FSH. Among them, 571 juvenile patients (JP) were admitted, which accounted for 4.6% of the total admitted patients.

Lockdown conditions may affect children’s nurturing care, including their health, nutrition, security and safety, early learning, and responsive caregiving, with long-lasting effects on their development that may be difficult to compensate for [6, 7]. Some studies identified a higher prevalence of mental health symptomatology among children during the COVID-19 pandemic than before [8, 9]. Based on the influence of the epidemic on the physical and mental health of JP, we conducted in-depth interviews with 16 parents of the JP in the FSH. The aim of present study is to explore the psychological experience of JP’s parents regarding the health of their children in the FSH, to aid in improving individual, humanized and practical medical care services.

## Materials and methods

### Study design

A descriptive phenomenological research method was conducted [10]. One-to-one, face-to-face and semi-structured interviews were adopted to explore the psychological experience of JP parents in the FSH.

### Sample and recruitment

From April 9 to May 6, 2022, 16 parents of JP with COVID-19 were recruited from the National Exhibition and Convention Center (Shanghai, China) FSH using the method of purposeful sampling [11] (Table 1).

**Table 1:** Demographics and characteristics of parents of juvenile patients and of the juveniles

No	Sex	Age	Occupation	Education Level	Marital Status	Diagnostic Classification	Vaccinated(Y/N)	Juvenile’s sex	Juvenile’s age	Juvenile’s infection status	Juvenile’s Vaccinated(Y/N)
1	M	36	Freelancer	Middle school	M	asymptomatic	Y	M	7	asymptomatic	Y
2	F	43	Administrator	Undergraduate	M	asymptomatic	Y	M	6.5	mild	Y
3	F	34	Data analyst	Undergraduate	M	mild	Y	F	5	mild	N
4	F	45	Editor	Doctor	M	asymptomatic	Y	M	12	asymptomatic	Y
5	F	32	Salesperson	Junior college	M	asymptomatic	Y	M	2.7	mild	N
6	F	41	Engineer	Undergraduate	M	asymptomatic	Y	F	12	mild	Y
7	F	32	Company staff	Undergraduate	M	mild	Y	F	6	mild	N
8	F	44	Administrator	Junior college	M	asymptomatic	Y	M	12	asymptomatic	Y
9	M	39	Real estate developer	Undergraduate	M	mild	Y	F	10	mild	Y
10	F	40	Housewife	Junior college	M	mild	Y	F	5.5	mild	Y
11	F	43	Housewife	Elementary school	M	asymptomatic	Y	F	3	mild	N
12	M	38	Landscape designer	Undergraduate	M	asymptomatic	Y	M	6	asymptomatic	N
13	F	41	Accountant	Undergraduate	M	asymptomatic	Y	F	13	mild	Y
14	F	26	Housewife	Middle school	M	mild	Y	M	2.8	mild	N
15	F	36	IT specialist	Master	M	mild	N	F	0.9	mild	N
16	F	36	Housewife	Elementary school	M	mild	Y	M	9	mild	Y

The inclusion criteria were as follows. (1) All the JP and their parents were isolated in the same cabin concurrently with positive nucleic acid detection for COVID-19; (2) Given that JP over the age of 15 acted independently and were rarely accompanied by parents, participants were parents of JP under 14 years of age; (3) Participants had good verbal skills to ensure the interview process went well; (4) Before interviews, participants were fully informed of the purpose and procedure of the study by Interviewer. There was no incentive for participants to participate. They did so voluntarily.

According to the inclusion criteria, the JP’s parents were preliminarily selected from the list of scheduled discharges in our unit. The JP’s parents were divided into four groups according to their children’s ages: Group A (0–3 years old), group B (3.1–6 years old), group C (6.1–10 years old), group D (10.1–14 years old). Participants were interviewed individually. The sample size was not determined until the data of interviewees were saturated with no new topics. Sixteen people were interviewed in this research. Before the interview, a demographic and characteristic form of JP and their parents was filled out. The details are found in the Table 1.

### Data collection

Based on the literature review, a preliminary interview outline was drawn up after discussions with members of the research team and three expert consultants in the fields of nursing, management and psychology. We conducted a pre-interview with two participants. After discussion and revision by the research group, the final interview outline was determined as follows: (1) How do you view isolation in the FSH caused by the epidemic? (2) How do you think this epidemic has affected you and your child? (3) What are your major concerns/difficulties since the isolation in FSH? (4) Do you have any ideas or suggestions on isolation in the FSH? (5) What concerns do you have after you are discharged from the hospital?

The interview was conducted by the same researcher as the one who conducted the pre-interview. In order to avoid interruptions and personal privacy disclosure, the interview was conducted in a study room in the FSH. Interview time was scheduled with the interviewee in advance. No more than three participants were interviewed each day for 20–30 min. Before the interviews, the interviewees were informed that the interviews would be recorded by the interviewer and signed informed consent forms. All participants agreed to be recorded. During the interview, the interviewees' concerns were respected. The interviewees' views and logical structure of answering questions were respected, so that interviewees thoroughly revealed their views on their own rather than being coaxed to do so by the interviewer.

Interviewees' interests were respected and sensitive topics were avoided. Interviewees' questioning and refusal were also respected. The interviewer was compassionate, understanding and interested in the shared experience. Interview skills such as listening and questioning were performed to assess the interviewees' real feelings. An open interview was adopted to help the interviewer follow the interviewees' thinking. Leading language was avoided in the interviews. The interviewer helped participants describe their experiences without leading the discussion. Interfering, interrupting and forcing were avoided in the interviews. The interviewer suspended prejudice and kept an objective attitude. Inducement and judgment were avoided. The numbers N1 ~ N16 were used to avoid interfering with the interviewee’s privacy.

### Ethical consideration

This study was approved by the Ethics Committee of the Xinqiao hospital of Army Medical University (No. 2022–201-01), conforming to the ethical guidelines of the 1975 Declaration of Helsinki (6th revision, 2008). Interviewees were informed of the purpose and significance of the study. The interviewer obtained written informed consent from the participants before enrolment. Participants were assured their personal data would be strictly confidential.

### Data analysis

Data collection and analysis were carried out simultaneously. The Colaizzi seven-step analysis method was used [12, 13]. At the end of each interview, the researchers converted the recording to text through the text assistant APP [14]. Then, the text was checked by listening to the recording repeatedly, modifying it precisely to correspond with the recording. Before the final version of the interview text was formed, the text document was returned to the interviewees for confirmation through Wechat, a social APP for smartphone [15]. After the interview, the text was analyzed and crucial, unique, recurring and meaningful language units were coded (the specific description of interviewees was marked). The sub-themes and themes were summarized step by step. The interview continued until the data of interviewees reached saturation.

## Results

**Fig. 1:**
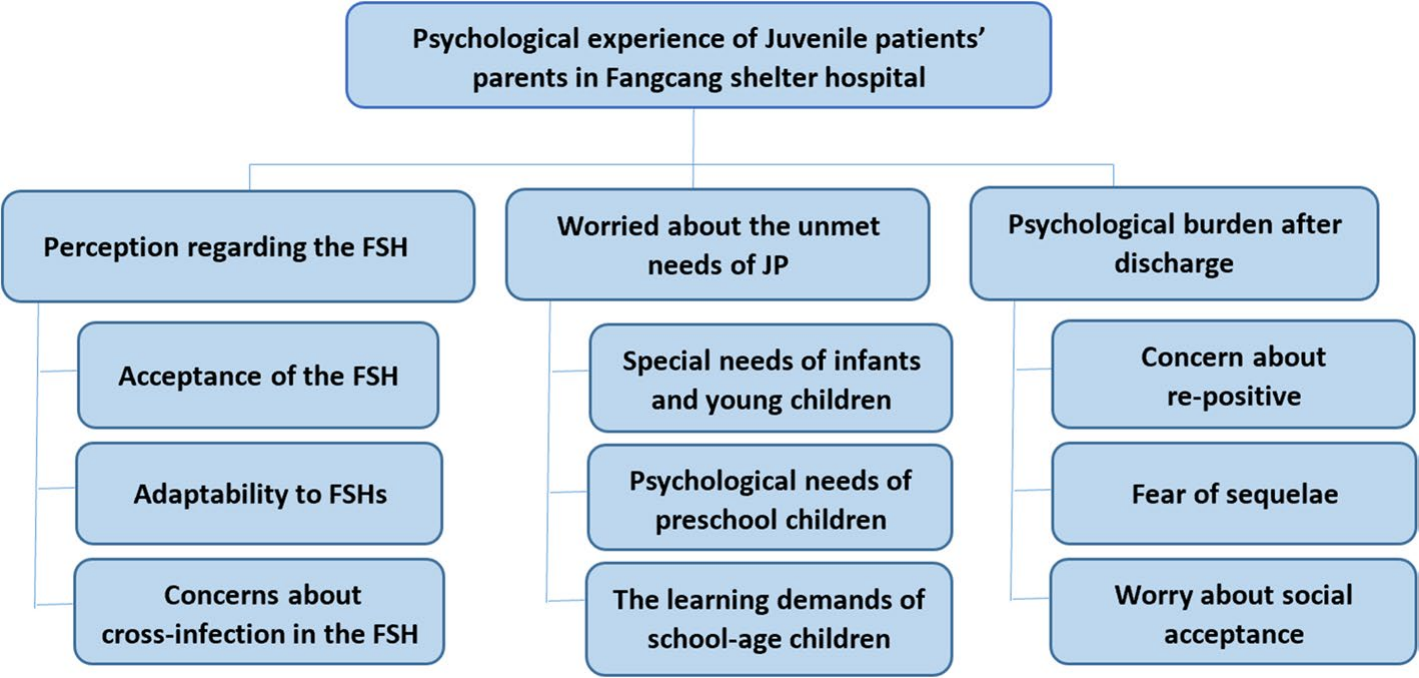
Key themes and subthemes tree

Three key themes emerged from the analysis of interviews: perception regarding the FSH, worried about the unmet needs of JP, and psychological burden after discharge. All of the key themes and subthemes of the psychological experience of the Juvenile patients’ parents were summarized in Fig. 1.

### Theme 1 Perception regarding the FSH

#### Acceptance of the FSH

Most parents believed that it was necessary to establish FSHs where professional medical staff could deal with the symptoms of their children in a timely manner; the asymptomatic carriers could be centrally isolated; and the epidemic could be effectively controlled.

N10: "At first, I didn't think it would be so serious. I thought I was safe at home because I had been vaccinated. But after my child was infected, we were very worried and didn't know how to deal with it. After being admitted in the FSH, we felt at ease.”

*N9: “The country's overall policy was favorable. Omicron was very contagious. It was right to set up FSHs. Personally, I thought it was an issue of control. We should isolate effectively and work together to control the epidemic.”*

#### Adaptability to FSHs

Some parents reflected that environmental changes, lighting intensity and other factors might lead to poor sleep quality of children. During the early stage, infants and young children were afraid, unable to adapt and wanted to go home, whereas preschool children and school-age children had stronger adaptability. After family bed placement, material support, environmental improvement and medical staff care, the vast majority of children and parents quickly adapted to the situation.

*N12: "The light is too bright to sleep well. As the child left his mother and the environment changed, he was a little uncomfortable.”*

*N3: "It's better than my expectation. At the beginning, I might have felt a little uncomfortable, but my various needs were met. I could understand the current conditions. It was well organized. "*

*N6: "There was some negative news from various aspects. I was worried at first. But, at this time, the whole family was quarantined in the FSH, and the supplies were sufficient. The medical staff and volunteers worked very hard, and the government management had been increasingly well-organized. I felt less afraid on the basis of this experience."*

*N10: “Novelty and excitement was shown on account of the mild symptoms of the young children. Consequently, they could adapt well to the FSH. In addition, there was a favorable atmosphere and a sense of security here. We felt no pressure because of you doctors and nurses.”*

#### Concerns about cross-infection in the FSH

In this study, the biggest concern facing all parents in the FSH was cross-infection. Some parents worried that their children were infected repeatedly.

*N12: "If my child turned negative while I was still positive, he must stay in FSH with me because no one could take care of him in the family. I was worried that he might suffer repeated infection. "*

*N14: "I dared not take risks to let him contact anyone else or play with other children because of the concern about cross-infection ".*

### Theme 2 Worried about the unmet needs of JP

#### Special needs of infants and young children

In this interview, there were four parents of infants, the youngest of whom was 11 months old. The situation regarding infants was particularly prominent in terms of life and security needs. Although some necessary supplies for infants were prepared, the personalized needs of infants could not be fully met in the FSH. The inability to meet the nutritional needs of children with respect to, for instance, various fruits or supplementary foods was a worry. In addition, because of their young age, they lacked the sense of security of living with thousands of people in the same hall.

N15: "The child was too young to face too many strangers. Dietary supplements and various fruits could not be provided in the FSH. Besides, it was not convenient for me to take a bath or go to the toilet when I was taking care of the baby alone. I was worried."

#### Psychological needs of preschool children

There were 4 parents of preschool children in this interview. All parents of children in this age group considered that isolation had little effect on their children’s studies since they were young. However, they were lonely and found everything around them strange. They longed for freedom to make friends in the FSH.

*N12: "At the beginning, he was afraid, but later, he felt better after adaptation. He was curious about various things in the FSH and wanted to play with others around them. However, he was still eager to go back to the kindergarten."*

#### The learning demands of school-age children

There were 8 parents of school-age children in this interview. The oldest child was 13 years old. One of the children was about to graduate from elementary school. It was supposed by parents of senior students that the epidemic had a great impact on their children's study, and that a gap could be observed between online courses and offline teaching. In contrast, parents of junior students thought that the epidemic had little impact on their children's learning, and online classes in the learning room of FSH could meet their learning requirements.

*N14: " Epidemic had a big impact on school work, which led children not to attend classes normally. The poor online learning efficacy might be described as being too many people, too much noise and instable Internet signal in the FSH."*

*N2: "My son was in grade one. His school was closed due to the epidemic, but he could go on with his courses in the FSH. Parents could help children with junior grade schoolwork. The head teacher promised to make up the missed lessons for him when he went back. So, there was little impact on his studies.”*

### Theme 3 Psychological burden after discharge

#### Concern about re-positive

According to China's COVID-19 Diagnosis and Treatment Protocol (Trial Version 9) [16], the criteria for discharge from the FSH were: negative nucleic acid tests for COVID-19 two consecutive times (with an interval of at least 24 h between samples), normal body temperature for at least 3 days, and obvious remission of respiratory tract and other clinical symptoms. Faced with the highly contagious Omicron variant strain, 10 parents in the interview were worried about re-positive nucleic acid detection after discharge.

*N5: "It was said that some individuals who underwent re-positive nucleic acid detection might come back to the FSH again after discharge. I was really concerned about my child with re-positive nucleic acid detection."*

*N9: "It was not clear what led to the re-positive nucleic acid detection. I was concerned about the re-positive. I didn’t know what I could do after being re-positive. I was afraid that the children’s ability to study would be affected by re-entry into the FSH."*

#### Fear of sequelae

Some parents reacted with worry in response to the unknown threat of COVID-19 to humans as well as public opinion on the Internet. In the interview, four parents were concerned about sequelae after their children were infected with COVID-19.

*N3: "I was worried about the negative impact of COVID-19 on the body, leaving sequelae for my child, giving rise to her poor physical quality in the future, retarding her growth and development."*

*N14: "My child was still young and had not been vaccinated against COVID-19. I didn’t know if there would be sequelae after being infected with COVID-19. It was said on the Internet that people who were infected with COVID-19 could not have children. Was this true? ".*

#### Worry about social acceptance

Under the context of the unknown and ever-changing COVID-19, the public may lack a sense of psychological security even with negative nucleic acid detection. In the interview, four parents referred to the problem of social acceptance.

*N6: "I was worried about the pressure and fear of my neighbors after discharge from the hospital. My child would be isolated by the other children in the community."*

*N8: "It was claimed that some companies would not accept these individuals who had been infected with COVID-19 because they were prone to being discriminated against by others. I was afraid the work loss in future."*

## Discussion

### Planning facilities for JP in advance before FSH construction

FSH has the task of undertaking large-scale natural disaster rescue and mass infectious disease treatment. It played a critical role in disaster relief after several natural disasters and early stage of resistance to COVID-19 [17]. However, the Omicron wave in Shanghai infected tens of thousands of people, and there were a large number of people in the FSH. The change of living conditions and environment may lead to maladaptation of many patients. Even more to the point, the JP were a vulnerable group, because their adjustment and feedback mechanism regarding stress events was immature, which may also contribute to maladaptation. In the present study, it was indicated that potential factors such as environmental changes and lighting intensity could lead to children’s poor sleep quality. The needs of children vary from age to age. Consequently, parent–child cabins should be constructed to meet the needs of JP during the construction and design of FSH.

A recent study showed that dissatisfaction with some of the facilities and services at the hospital were some of the major challenges those parents experienced while seeking care for their children [18]. A healthcare environment which enables intellectual, emotional, social and motor development is required for both children and adolescents. These requirements should be implemented in hospitals [19]. The hospital's built environment could improve the mood, functioning, and perceived quality of care for parents. Child-centered and family-friendly design features, such as light dimmers control panels, more privacy, less noise, more amenities, appear to be associated with children's quality of sleep and decrease in anxiety during hospitalization [20]. Moreover, facilities with activities for adolescents to go to in separate spaces are necessary [21].

Future issues to consider in the design of new FSHs should systematically address methods to optimize the environment for JP. It should include setting up a big bed, baby bed, safe bed, baby seat, children's tableware [22]. Each sheet unit should be equipped with curtains to strengthen privacy protection for shading in cloth or include a knob, which could control and adjust the light intensity independently in their own area [23]. For children under 6 years old, an activity area should be set up in zones with air circulation, equipped with toys and game props that can be disinfected repeatedly [24].

During each activity, everyone needs to wear a mask, wash their hands, keep a distance greater than 1 m. For school-age students, the learning room should be equipped with desks, chairs, lamps that can be disinfected repeatedly and networks with stability and high speed. Each desk and chair should be kept at a distance of more than 1 m. These public facilities should also be used once per person and disinfected after use to avoid cross-infection [25, 26]. Accordingly, excellent FSH facilities for the requirements of JP could promote the adaptability of JP.

### Provide humanistic care according to the needs of JP in different ages

Different from general hospitals, FSH was a social space which should provide prerequisite emotional support and social participation for patients [27]. In China, FSH are considered as a community of patients, who are isolated from the COVID-19-negative population but support each other and participate in social activities. Health workers provided emotional support in addition to medical care, and community activities included eating together, watching television, dancing, reading, and celebrating birthdays [27–29].

The interviewees in this study expressed the different needs of JP found in different ages. Therefore, personalized care should be carried out according to the needs of JP found in different ages in the FSH. It should provide children with a variety of food to ensure that nutritional needs are met, such as children's meals, baby formula, milk, eggs, fresh foods, pulses, vitamin-a rich fruits and vegetable and others [30]. As well, appropriately sized masks are needed for children. It should pay attention to children's mental health. Group or individual counseling should be conducted by professional psychologist [31]. It should conduct children's activities, such as COVID-19 epidemic knowledge context, story-telling session, crafts, calligraphy, painting, games, etc. In addition, thematic activities, for instance Labour Day, Mother's day, could be conducted to encourage children to take the initiative in expressing their emotions and reducing children's anxiety [32]. Students with learning needs regarding printed learning materials, tutoring, etc., should be helped [33]. Patient volunteers with education experience could also be invited to participant in organizing activities and tutoring for children [34–36]. These recommendations would help to promote the physical and mental health of JP in FSH during the pandemic.

### Promote public scientific cognition through health education strategies

Interviewees in this study were concerned about cross-infection in the FSH, re-positive after discharge, sequelae and social discrimination. As a vulnerable group ineligible for COVID-19 vaccination, children under 3 years of age were at high infection risk and needed protection. Previous studies have demonstrated that the incidence of Omicron variant SARS-COV-2 infection in children under 5 years old was 6–8 times that of Delta variant, while the incidence of omicron infection in children between 0 and 2 years old was higher than that in children between 3 and 4 years old [37, 38].

However, there are some knowledge gaps between medical staff and the public. Therefore, offline and online methods, such as lectures, brochures, picture scrolls, videos, audio etc., should be provided by medical staff to promote the public's scientific awareness of Omicron [39]. (1) It should guide the JP to take active protective measures, such as wearing masks, keeping a distance of 1 m, washing hands frequently, and proper cough etiquette to avoid cross infection [40, 41]. (2) It should explain the issues related to re-positive and sequelae to JP parents scientifically [42]. Studies have shown that most re-positive patients with mild or asymptomatic symptoms had low viral load and generally low infectivity risk [43].

Additionally, it is necessary to guide parents not to believe reports about sequelae from unknown sources on the Internet blindly and refer to information from sufficient scientific evidence [44, 45]. (3) In terms of the social acceptance of COVID-19 infection, infectivity of recovered COVID-19 patients and relapse cases should be publicly declared; however, this results in the same problem of resuming work and study as the original infection. As a result, policies should be drawn up to protect patients’ rights, reduce public panic, and enhance social support and social acceptance [46].

### Limitations

There were several limitations in this study. Firstly, this study was conducted in a unit of FSH during the Omicron wave of COVID-19 pandemic in Shanghai, China. As different countries, regions, backgrounds, diseases, and samples may influence the results, the generalizability of the results in other contexts needs further demonstration. Secondly, the depth and time of interviews may have been affected by the protective measures that researchers were required to take, they had to wear personal protective equipment and stay 1 m apart from each other. Thirdly, the interviews were not conducted in English. Thus, the responses of the interviewees, translated for publication, may not fully represent the original intentions of the interviewees in making their statements.

## Conclusion

As the infection and hospitalization of those infected with Omicron variant SARS-COV-2 reached a record level, JP as a vulnerable group requires increasing attention from the family and community. Specifically, much focus should be put on the prevention and protection of JP, and the treatment and rehabilitation of them. This study conducted in-depth interviews with parents of JP to understand their experience in the FSH, including "perception regarding the FSH ", "worried about the unmet needs of JP ", and "psychological burden after discharge". It proposes that juvenile facilities should be planned before FSH construction; the needs of JP of different ages should be paid attention to in providing humanistic care; and promote public scientific cognition through health education strategies. To make it attainable, affordability is one aspect on which FSH should be focusing. The outcomes and suggestions of our present study may be beneficial to improve the management of FSH, thereby relieving the psychological pressure of JP and their parents effectively, and facilitating the adaptability, and the physical and mental health of JP.

## Data Availability

All the research data is available on request from the corresponding author. For communication email luochunmei@tmmu.edu.cn.

## Abbreviations

COVID-19: Coronavirus disease 2019
SARS-CoV-2: Severe acute respiratory syndrome coronavirus 2
FSH: Fangcang shelter hospital
JP: Juvenile patients
